# Inadvertent Stone Migration During Pneumatic Lithotripsy: Still a Conundrum in the 21st Century

**DOI:** 10.7759/cureus.10521

**Published:** 2020-09-18

**Authors:** Ali Akbar Zehri, Miten Patel, Philip B Adebayo, Athar Ali

**Affiliations:** 1 Surgery, The Aga Khan University, Dar Es Salaam, TZA; 2 Neurology, The Aga Khan University, Dar Es Salaam, TZA

**Keywords:** anti-retropulsion device, ureteric stones, pneumatic lithotripsy, retrograde stone migration, stone-free rates, ureteroscopy

## Abstract

Currently, an ideal gadget to stop retrograde stone migration remains a holy grail, and the hunt for such a device is still ongoing in the 21st century. The quest for an ideal instrument is driven by the need to reduce cost, minimize ancillary procedure rates, reduce the device's operative time, and improve the stone-free rate. The purpose of the present review is to provide an update on the use of preventive measures that are used to stop retrograde stone migration during pneumatic lithotripsy for ureteric stone management.

## Introduction and background

Ureteroscopic pneumatic lithotripsy for the treatment of ureteric calculi is quite a common modality [1]. However, proximal migration of the calculi during lithotripsy has been reported to range from 3-48% in the literature [2]. The migration depends on various factors; it can be triggered by the jet of irrigation fluid, type of energy source used for intracorporeal lithotripsy, location of the calculus in the ureter and the degree to which it is impacted there, as well as the degree of proximal ureteral dilation [3]. Electrohydraulic and pneumatic lithotrites are more prone to retrograde migration of calculi compared to other devices like ultrasonic or holmium:yttrium-aluminum-garnet (Ho:YAG) lasers [4]. However, in the case of Ho:YAG lasers, the rate of stone retropulsion increases proportionally to the rise in pulse energy used or the diameter of the optical fiber. The proximal migration of the calculi is more when the stones are smaller or when the diameter of the proximal ureter is larger, e.g., in hydronephrosis [3].

The migration of stone fragments when performing lithotripsy results in increased morbidity and cost burden to the patient [2,5]. Retrieval of these fragments may require various additional procedures. These include flexible ureterorenoscopy, further fragmentation, need for special retrieval apparatus, ureteric stenting, or in some cases, extracorporeal shock wave lithotripsy (SWL) [3,4]. Moreover, these fragments act as a nidus for infection or further growth or may lead to colic. Various devices have been developed to ease the extraction of ureteric calculi and prevent their retrograde migration during lithotripsy. However, these devices are far from perfect with each carrying its own limitation and varying success rates [6-8]. There are a number of accessory instruments, which have come up recently, to be placed above a calculus so as to prevent migration of fragments proximally during ureteroscopy. They also facilitate the extraction of fragments upon the removal of these devices [3,4].

The Stone Cone (Microvasive; Boston Scientific, Marlborough, MA) is a coupling nitinol wire that is about 0.43-mm into a 3F polytetrafluoroethylene (PTFE) sheath. The nitinol has a specialized tip that is shaped in concentric coils. When these coils are open and placed proximal to the stone, they prevent any migration of the stone or its fragments during lithotripsy [9]. Another option would be the use of 2cc of 2% lidocaine jelly that is instilled just proximal to the stone using a 5-cc syringe to avoid migration of fragments to the proximal ureter [8]. The PercSys Accordion (Percutaneous Systems, Palo Alto, CA) is a unibody device of about 2.9F that has a multi-fold polyurethane film backstop that can provide a 7-mm barrier when fully deployed [10].

A number of other devices are also reported in the literature; however, of these occlusion devices, the Stone Cone, NTrap (Cook Urological, Spencer, IN), and lidocaine jelly installation proximal to ureteric stone probably have the most successful profiles [11,9]. In this review, we compared the safety and efficacy of these devices with lidocaine gel in minimizing retrograde migration of calculi and extraction of fragments during ureteroscopic lithotripsy.

## Review

1. Methodology

A narrative literature review was performed using MEDLINE®, Google Scholar, and The Cochrane Database of Systematic Reviews (CDSR) to identify relevant studies. Searches were restricted to publications in English and procedures in the adult population from 1994 to December 2019. Separate searches were performed with the following search terms: "anti-retropulsion device", "ureteric stones", "pneumatic lithotripsy", "retrograde stone migration", stone-free rates, and "ureteroscopy". Article selection proceeded according to the search strategy based on Preferred Reporting Items for Systematic Reviews and Meta-Analyses (PRISMA) criteria. Only those studies comparing different anti-retropulsion devices during ureteroscopy to prevent retrograde migration were included for further screening. Cited references from the selected articles retrieved in the search were also assessed for significant papers. Conference abstracts were not included because sufficient details for the study were not available in the abstracts. All authors and one independent reviewer completed this process, and all disagreements were resolved by reaching a consensus on them.

2. The problem: stone migration during ureteroscopy

One of the major challenges a urologist faces during ureteroscopic lithotripsy is stone migration or retropulsion. There are several factors that play their part here. It could be due to the energy transmission into the calculi used to fragment the stone during lithotripsy or the irrigation flow used during ureteroscopy. Inadvertent push has been commonly observed with pneumatic lithotripsy, ranging from 3-48% as reported in the literature; however, this varies based on the stones' localization [2,12,13]. The risk of retrograde migration during Ho:YAG laser lithotripsy has been shown to be lower than other modalities [3]. There is a considerably higher risk of proximal migration when the ureter proximal to the stone is dilated. The risk of migration also increases the more proximal you are and depends on the operating surgeon's experience with the procedure [11]. Higher volume centers with good experience in ureteroscopy have been able to achieve migration rates as low as 4-7% [9].

Different studies have shown varying stone migration rates, with higher rates for stones in the proximal ureter when pneumatic intracorporeal lithotripsy was used. A study by Knispel et al. found that they had a migration rate of 40% for stones in the proximal ureter vs only 5% when the calculus presented in the distal ureter [12]. Robert et al. also reported a 48% migration rate of stones in the proximal ureter [2]. Recently, Chow et al. noted that this risk was not eliminated with newer techniques, such as laser lithotripsy or flexible ureteroscopy. Even these techniques had 25% retrograde migration for proximal ureteric stones [14]. Most of the migrated stones or residual fragments need secondary procedures like SWL or another ureteroscopy. Moreover, these residual fragments act as a nidus for recurrent stone growth, renal colic, or even infections. Additionally, they increase costs and morbidity rates among patients [3,15,16].

3. Various options: merits and demerits

An arsenal of devices and strategies have been developed in recent years to prevent proximal stone migration and aid extraction of stone fragments. These include Parachute (Microvasive; Boston Scientific), Lithocatch stone baskets (Microvasive; Boston Scientific), Lithovac (Microvasive; Boston Scientific), Passport Balloon (Microvasive; Boston Scientific), NTrap (Cook Urological), and the Stone Cone (Microvasive; Boston Scientific) [8,9,15-17]. These devices have a number of limitations that prevent their regular usage in lithotripsy. A major limiting factor is a need for all these devices to be left in the working field, which is already limited, thereby limiting maneuverability of the scope. The Lithocatch and Parachute are basket mesh-based models. The disadvantage of this model is that their basket may unintentionally trap fragments, making it difficult to disengage them and may potentially cause ureteral injury [11]. Studies have demonstrated that the ball-bearing ability of the Parachute to retrieve is similar to that of a basket [18]. Studies have also reported that using the 0.038-inch Passport Balloon compromises the success rate of advancing a flexible ureteroscope past the stone [15].

Another effective device to retrieve smaller fragments is the Dretler stone cone. This device has an additional safety feature: its coils begin to unwind in case the volume of fragments in the cone exceeds the safety limit [19]. Moreover, the Dretler rotates and unwinds when traction is applied to it. This process produces lower traction as compared to simple traction.

Among other strategies used to prevent proximal migration is the use of baskets. There are some reported cases of wire or basket damage during lithotripsy. Removal of these damaged baskets or parts is very challenging even for the most experienced hands. Similarly, obstructing balloons may be used to prevent proximal migration. However, they work best in a system that is not dilated. If the proximal system is dilated, then the balloon may be too small to work effectively. Damage to the balloons by lithotripters has also been reported.

The Stone Cone and NTrap are stone trapping devices of more recent origin that form proximal barriers to prevent stone migration. They have been shown to be effective in many studies. However, like other devices, they too have some limitations. The NTrap device unwinds by the force of the lithotripsy or if higher flows of irrigation are used. They too may not be able to fully occlude the ureters in cases of significant ureteric dilation beyond the diameter of its barrier.

4. Cost implications

Retrograde migration of stone fragments is associated with several complications, ranging from prolonged operative time to the need for supplementary procedures, all increasing costs to various degrees. Most of these devices are disposable, hence one must be able to fairly justify their added cost vis-à-vis benefits. A study by Ursiny et al. reviewed occlusion devices including BackStop, NTrap, the Stone Cone, and lidocaine jelly, and found that these devices would be cost-effective when the proximal migration rate was greater than 6.3%. However, they reported that these data should be interpreted with caution, because, in reality, there would be various treatment options for the retropulsed fragment, such as, from observation, the use of flexible ureteroscopy or perhaps a secondary procedure, most often SWL or ureteroscopy. The limitation of their study was the assumption that all patients with proximal stone migration of stone fragment would undergo secondary procedures. Though the cost implications of each treatment modality would vary, the cost of each modality could easily surpass that of these devices as shown in Table 1 [19,20].

**Table 1:** Anti-retropulsion devices with cost

Device	Manufacturer	Cost
NTrap	Cook Urological	$100
PercSys Accordion	Percutaneous Systems	$325
BackStop	Boston Scientific	$50
Parachute	Boston Scientific	$99
Lithocatch	Boston Scientific	$80
Passport Balloon	Boston Scientific	$230
Stone Cone	Boston Scientific	£215
Lidocaine gel	AstraZeneca Pharma	$3

5. Lidocaine gel instillation

Instillation of high-viscosity lidocaine jelly proximal to ureteral stones to prevent stone migration is another technique that has been reported. Once the procedure is complete, the jelly can be washed off with irrigation or, at times, we can even let it dissolve on its own. There have been some studies, including one by Zehri et al. [8], that showed significantly higher stone-free survival rates with the use of lidocaine gel when compared to placebo. However, a study by Sen et al. could not show that lidocaine was superior to other occlusive devices. One must also keep in mind that this technique also has its drawbacks, such as the difficulty to wash off the jelly with irrigation and the jelly obscuring the view of the ureteroscopy [20].

## Conclusions

The quest for an ideal anti-retrograde migration device still continues in the 21st century. Each tool has its own drawbacks and benefits, often necessitating the use of multiple gadgets to achieve successful results. The ideal device should be easy to place, overcome fragment migration, and allow for the passage of intracorporeal lithotriptors, guidewires, and stents following stone fragmentation. More importantly, it should be cost-effective for the patient as compared to ancillary procedures. Xylocaine jelly instillation is a low-cost method and it is easily available. It is associated with high stone-free rates and can reduce auxiliary procedure rates. However, further high-volume studies are required to justify its benefits.
